# Predictive significance of the hemoglobin, albumin, lymphocyte, and platelet score for radiation pneumonitis in lung cancer patients: a respective comparative study with dosimetric parameters

**DOI:** 10.3389/fonc.2025.1605094

**Published:** 2025-06-04

**Authors:** Xiaoxuan Xie, Ming Pei, Meng Chen, Yun Zhou, Dunqiang Ren, Yongzhong Guo

**Affiliations:** ^1^ Department of Radiotherapy, Xuzhou Central Hospital, Southeast University Affiliated Xuzhou Central Hospital, The Xuzhou School of Clinical Medicine of Nanjing Medical University, Xuzhou Clinical School of Xuzhou Medical University, Xuzhou, Jiangsu, China; ^2^ Shanghai Key Laboratory of Forensic Medicine, Key Laboratory of Forensic Science, Ministry of Justice, Shanghai, China; ^3^ Institute of Forensic Science, Xuzhou Public Security Bureau, Xuzhou, Jiangsu, China; ^4^ Department of Respiratory and Critical Care Medicine, The Affiliated Hospital of Qingdao University, Qingdao, Shandong, China; ^5^ Department of Respiratory and Critical Care Medicine, Xuzhou Central Hospital, Southeast University Affiliated Xuzhou Central Hospital, The Xuzhou School of Clinical Medicine of Nanjing Medical University, Xuzhou Clinical School of Xuzhou Medical University, Xuzhou, Jiangsu, China

**Keywords:** hemoglobin, albumin, lymphocyte and platelet score, radiation-induced lung injury, lung cancer, radiotherapy, malnutrition

## Abstract

**Introduction:**

Inflammatory response and nutritional status have been linked to adverse reactions of radiotherapy. The hemoglobin, albumin, lymphocyte, and platelet (HALP) score, associated with both inflammation and nutrition, can effectively predict prognosis in various cancers. However, its role in predicting radiation pneumonitis (RP) among radiotherapy patients remains unclear, and further investigation is needed to elucidate it.

**Methods:**

The general clinical data of lung cancer patients who underwent radiotherapy between January 2021 and October 2024 were retrospectively collected. RP was graded in accordance with the Common Terminology Criteria for Adverse Events (CTCAE) version 5.0. Predictive factors for RP were identified using LASSO and multivariate logistic regression analyses, and a nomogram was subsequently developed based on these factors. The predictive performance of the nomogram was comprehensively evaluated using the area under the receiver operating characteristic (AUROC) analyses, calibration curve, and decision curve analysis.

**Results:**

A total of 396 patients’ data were analyzed (development cohort: 301; temporal validation cohort: 95). Multivariate logistic analysis revealed that the HALP score and lung volume receiving ≥5 Gy (V5) were independent predictors of symptomatic RP, and regarding severe RP were HALP, V5, albumin, and hemoglobin. The AUROC values of the HALP score were 0.77 (95% CI: 0.72–0.83) and 0.83 (95% CI: 0.76–0.90) for predicting symptomatic and severe RP. The integrated HALP-V5 model exhibited excellent predictive ability both in symptomatic RP (AUROC: 0.84; 95% CI: 0.79–0.89) and severe RP (AUROC: 0.89; 95% CI: 0.83–0.94), with high predictive accuracy and clinical utility.

**Conclusion:**

HALP can be employed as a promising independent predictor of RP in lung cancer patients undergoing radiotherapy, and the combination of V5 can further improve prediction accuracy.

## 1 Introduction

Lung cancer, accounting for almost 2.5 million new cases and over 1.8 million deaths in 2022, remains the most commonly diagnosed cancer and the leading cause of cancer death worldwide (1). Over half of lung cancer patients are diagnosed at advanced stages, rendering curative treatment often unfeasible (2). In this context, radiotherapy has become an indispensable component of multidisciplinary treatment for lung cancer. Over 70% of patients have evidence-based indications for radiotherapy during their care, ranging from curative treatment to symptom palliation (3). However, the development of radiation-induced lung injury (RILI), manifested as early radiation pneumonitis (RP) and subsequent irreversible pulmonary fibrosis, has become a critical obstacle to effective cancer control and patient survival. Although modern radiotherapy technologies have achieved remarkable progress in substantially reducing radiation exposure to normal lung tissue, the clinical incidence of RILI remains persistently high. Current literature demonstrates considerable variability in reported rates of RP, which can be as high as 58% (4). This substantial discrepancy is due to the numerous risk factors and the complex interplay of the diverse pathophysiological mechanisms involved (5–7). Of particular clinical concern, approximately 10%–20% of patients present RILI-related signs/symptoms of varying severity, with potentially progressing to life-threatening complications (6).

The lack of specific clinical symptoms or early imaging changes underscores the significance in identifying reliable biomarkers for early prediction and diagnosis of RILI (8, 9). Although many predictors or models for RP prediction based on diverse features have been proposed (5–7, 10, 11), substantial challenges remain in achieving clinical translation. Moreover, there is a paucity of clinical medications or interventions capable of reversing pulmonary fibrosis (4, 12). Therefore, timely and effective prediction of RILI in its RP stage represents major challenge in thoracic radiotherapy when achieving optimal tumor control.

The pathogenesis of RP is multifactorial interaction and incompletely understood. The inflammatory responses triggered by ionizing radiation, which contribute to tissue remodeling and subsequent fibrosis, are widely recognized as a predominant factor for RP development (4, 6, 13, 14). Concurrently, extensive research demonstrates the importance of nutritional status in modulating the adverse effects of radiotherapy (15–17). Despite the established links among inflammation, malnutrition, and RP, the clinical utility of inflammatory-nutritional biomarkers for RP prediction remains inconclusive, necessitating further investigation. The hemoglobin, albumin, lymphocytes, and platelets (HALP) score integrate markers of systemic inflammation and nutritional status (18), making it a promising candidate for predicting RILI. Although individual components of the HALP score link with RP risk in prior studies (16, 19–23), its predictive value for RP has yet to be explored.

Thus, we conducted this study to expand the value of HALP in predicting RP risk. Given the established predictive role of dosimetric parameters in RP (4, 7, 24), we compared the predictive performance of HALP with that of traditional dosimetric factors. Additionally, we attempted to develop a comprehensive nomogram integrating HALP and dosimetric parameters to visually stratify RP risk. To our knowledge, this represents the first systematic evaluation of the HALP score’s predictive value for RP. We expect that our findings can provide clinicians with valuable reference for identifying high-risk patients and informing clinical decision-making.

## 2 Methods

### 2.1 Patient selection and treatment

The medical records of lung cancer patients who received radiotherapy at Xuzhou Central Hospital (Jiangsu, China) between January 2021 and October 2024 were retrospectively collected from the hospital’s electronic medical records system (development cohort: January 2021–July 2024; validation cohort: August 2024–October 2024). Inclusion criteria were: pathologically confirmed lung cancer (any histology); clinical staging II–IV (American Joint Committee on Cancer, 8th edition); age ≥18 years at treatment; Eastern Cooperative Oncology Group (ECOG) performance status ≤2; life expectancy ≥3 months; treatment with intensity-modulated radiotherapy (IMRT) (definitive, adjuvant, or palliative) with/without other anti-cancer treatment; no prior history of thoracic radiotherapy; availability of clinical, laboratory, and dosimetric data; and at least six months of regular follow-up at 1, 3, and 6 months after radiotherapy completion. Patients with the following conditions were excluded: other conditions known to affect HALP levels (acute infection, hematological diseases, autoimmune disease, severe cardiovascular, hepatic, or renal impairment etc.); other interstitial lung diseases; incomplete data; inadequate radiation dose (<50 Gy) or premature discontinuation of radiotherapy; previous history of thoracic radiotherapy; or treatment with other radiotherapy techniques. The study was approved by the Ethics Committee of Xuzhou Central Hospital (XZXY-LK-20231102-0184) , and informed consent was waived because all collected information was derived from medical records without participant interaction.

With the advantages of better conformal dose distribution and a lower incidence of high-grade RP, IMRT has emerged as a primary radiotherapy option for lung cancer in recent years (2, 22). To minimize methodological bias, all enrolled patients received IMRT. All patients underwent radiotherapy using 6-MV photon energies. IMRT was planned using the Pinnacle 9.0 planning system and delivered via an Elekta Synergy linear accelerator. The target area and organs at risk are referred to the Radiotherapy and Oncology Group (RTOG) guidelines, and all treatment plans met standard certification requirements. The gross tumor volume, clinical target volume, and planning target volume (PTV) were defined following International Commission on Radiation Units and Measurements Reports 50 and 62. The treatment plan ensured that at least 99% of the PTV receives at least 95% of the prescribed dose, and the maximum dose to the PTV does not exceed 105%. Stringent dose-volume restrictions to normal tissues were as follows: lungs (lung volume receiving ≥5 Gy [V5] ≤70%, V20 ≤30%, mean lung dose [MLD] ≤15 Gy), heart (V30 <40%, V40 <30%), esophagus (V50 <30%), and spinal cord (maximum dose ≤45 Gy). All radiotherapy plans were certified according to standard requirements. The total radiotherapy dose ranged from 50–70 Gy, delivered at 1.8–2.0 Gy per fraction, once daily, 5 fractions per week.

Concurrent chemotherapy was administered using platinum-based doublet regimens: cisplatin or carboplatin in combination with paclitaxel, etoposide, or pemetrexed. Sequential chemoradiotherapy or radiotherapy alone was recommended for patients unsuitable for concurrent chemoradiotherapy. Additionally, consolidative immunotherapy was administered to some patients.

### 2.2 Data collection and outcome assessment

Data were obtained from medical records and the radiotherapy system. The following information was extracted: patient demographics (age, gender, body weight, height, smoking history, performance status, and comorbidities), cancer type, chemotherapy regimen, dosimetric variables (dose, V5, V20, MLD, and PTV), and laboratory values (albumin [ALB], absolute lymphocyte count [ALC], hemoglobin [Hb], and platelet [PLT] count) within one week before radiotherapy initiation. Regular follow-up examinations, including chest CT scans, were performed at 1, 3, and 6 months after radiotherapy. The data collection cutoff date was October 31, 2024.

Body mass index (BMI) was calculated as weight (kg) divided by the square of height (m) (kg/m^2^). The HALP score was calculated using the formula: Hb (g/L) × ALB (g/L) × ALC (/L)/PLT (/L) (18). Biological effective doses [BED] was calculated using previously reported formula: BED= nd(1 + d/(α/β)), with n = number of fractions, d = dose per fraction, and α/β = 10 Gy.

The diagnosis of RP was established based on a combination of clinical symptoms and chest CT findings. RP severity was graded according to the Common Terminology Criteria for Adverse Events (CTCAE) version 5.0 (**Supplementary Table S1**), with grade ≥2 defined as symptomatic RP and grade ≥3 as severe RP. The diagnostic and grading process for RP followed a three-phase protocol: an initial independent, dual-blinded evaluation by two researchers; resolution of disagreements through consensus with a third specialist; and, if disagreements still remained, joint review and finalization conducted by all three specialists.

### 2.3 Statistical analysis

Continuous variables were presented as median and interquartile range (25th–75th percentile), whereas categorical variables were reported as frequencies and percentages. Differences between groups were analyzed using the chi-squared or Fisher’s exact test, unpaired *t*-test, or Mann-Whitney U test, depending on appropriateness. Predictive factors for RP were selected using least absolute shrinkage and selection operator (LASSO) regression in high-dimensional data via 10-fold cross-validation to ascertain the optimal Lambda parameters. The selection of preliminary screening variables was determined by nonzero coefficients in the LASSO regression model. Only variables selected by LASSO were eligible for the subsequent multivariate logistic (enter method) regression and used to develop a monogram for predicting clinical diagnoses. To avoid the unstable and imprecise estimates of the coefficients, multicollinearity was tested to exclude highly correlated variables in the final multivariate logistic regression, with a variance inflation factor (VIF) >10 among independent factors indicative of multicollinearity. The Box-Tidwell test was employed to evaluate the linearity assumption between continuous predictors and the log-odds of the outcome. For variables showing a nonlinear relationship, quadratic transformations were applied, and the model was reconstructed accordingly. The Bootstrap resampling method (1,000 iterations) and temporal external validation were used to validate the nomogram. The performance of the nomogram was evaluated using receiver operating characteristic (ROC) curve analysis for discriminative ability, calibration curve analysis and the Hosmer-Lemeshow goodness-of-fit test for predictive accuracy, and decision curve analysis (DCA) for clinical utility. ROC analysis was also used to determine sensitivity, specificity, optimal cut-off thresholds (via maximum Youden index), and comparisons among various parameters. All analyses were performed using R version 4.2.2, with statistical significance set at *P <*0.05.

## 3 Results

### 3.1 Patient characteristics

**Figure 1:**
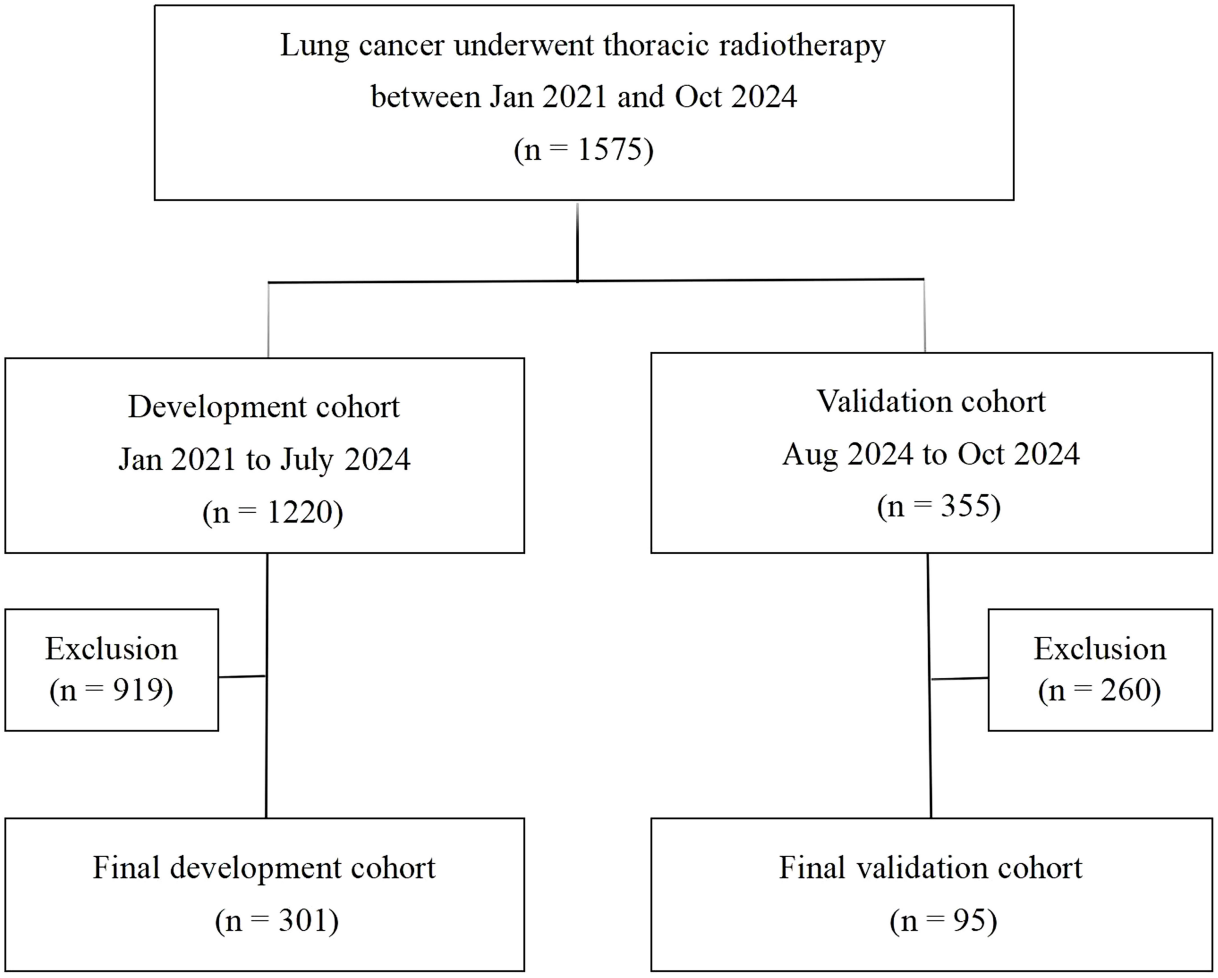
Flowchart of patient selection.

The study design and patient composition are illustrated in **Figure 1**. Following the inclusion and exclusion criteria, 301 patients were enrolled in the development cohort. The median age of the patients was 67 years (60–72), with the majority being male (74.4%). Adenocarcinoma was the most prevalent pathological type, accounting for 39.53% of cases, followed by squamous cell carcinoma (35.88%), small-cell lung cancer (21.26%), and other histology (3.32%). Patients with stage II, III, and IV represented 32.89% (99), 40.53% (122), and 26.58% (80), respectively. CCRT was administered to 133 (44.19%), sequential CRT to 144 (47.84%), and radiotherapy alone to 24 (7.97%) patients. Immunotherapy was administered to 74 patients (24.58%). Grades 0–4 RP were observed in 122, 82, 56, 37, and 4 patients respectively; no RP-related deaths occurred. **Table 1** details the main characteristics and treatment regimens of the development cohort patients. Significant differences in the presence of emphysema, BED, V5, V20, MLD, PTV, and HALP were observed between the non-symptomatic RP and symptomatic RP groups. Similarly, significant differences in V5, V20, MLD, PTV, and HALP score were noted between the non-severe RP and severe RP groups. Compared with the non-symptomatic and non-severe RP groups, the symptomatic and severe RP groups exhibited lower HALP scores and higher dosimetric parameters, including V5, V20, MLD, and PTV, respectively (**Table 1**)

**Table 1:** Baseline characteristics of patients with symptomatic and severe RP in the development cohort.

Variables	Overall (n = 301)	Symptomatic RP (n = 97)	Non-symptomatic RP (n = 204)	*P*	Severe RP (n = 41)	Non-severe RP (n = 260)	*P*
Gender				0.349			0.125
Female	77 (25.58)	21 (21.65)	56 (27.45)		6 (14.63)	71 (27.31)	
Male	224 (74.42)	76 (78.35)	148 (72.55)		35 (85.37)	189 (72.69)	
Age (years)	67.00 (60.00–72.00)	68.00 (61.00–73.00)	67.00 (59.75–72.00)	0.321	68.00 (63.00–73.00)	67.00 (59.00–72.00)	0.128
BMI (kg/m²)	23.44 (21.22–25.47)	23.03 (21.22–24.38)	23.44 (21.22–25.82)	0.186	23.31 (20.55–24.38)	23.44 (21.22–25.63)	0.435
ECOG PS				0.305			0.375
0	102 (33.89)	27 (27.84)	75 (36.76)		10 (24.39)	92 (35.38)	
1	138 (45.85)	48 (49.48)	90 (44.12)		21 (51.22)	117 (45.00)	
2	61 (20.27)	22 (22.68)	39 (19.12)		10 (24.39)	51 (19.62)	
Smoking				0.154			0.556
No	145 (48.17)	53 (54.64)	92 (45.10)		22 (53.66)	123 (47.31)	
Yes	156 (51.83)	44 (45.36)	112 (54.90)		19 (46.34)	137 (52.69)	
ILD				0.548			0.433
No	267 (88.70)	84 (86.60)	183 (89.71)		35 (85.37)	232 (89.23)	
Yes	34 (11.30)	13 (13.40)	21 (10.29)		6 (14.63)	28 (10.77)	
Emphysema				0.049			0.410
No	215 (71.43)	77 (79.38)	138 (67.65)		32 (78.05)	183 (70.38)	
Yes	86 (28.57)	20 (20.62)	66 (32.35)		9 (21.95)	77 (29.62)	
Histology				0.942			0.559
SCC	108 (35.88)	36 (37.11)	72 (35.29)		12 (29.27)	96 (36.92)	
AC	119 (39.53)	36 (37.11)	83 (40.69)		16 (39.02)	103 (39.62)	
SCLC	64 (21.26)	22 (22.68)	42 (20.59)		11 (26.83)	53 (20.38)	
Other	10 (3.32)	3 (3.09)	7 (3.43)		2 (4.88)	8 (3.08)	
Stage				0.610			0.671
II	99 (32.89)	31 (31.96)	68 (33.33)		13 (31.71)	86 (33.08)	
III	122 (40.53)	43 (44.33)	79 (38.73)		19 (46.34)	103 (39.62)	
IV	80 (26.58)	23 (23.71)	57 (27.94)		9 (21.95)	71 (27.31)	
ALC (10^9^/L)	1.78 (1.49–2.23)	1.59 (1.35–1.80)	1.90 (1.54–2.39)	<0.001	1.50 (1.06–1.66)	1.85 (1.50–2.33)	<0.001
Hb (g/L)	122.19 (14.97)	117.13 (13.55)	124.59 (15.04)	<0.001	112.37 (12.52)	123.73 (14.75)	<0.001
ALB (g/L)	40.85 (3.98)	39.66 (3.91)	41.42 (3.89)	<0.001	37.77 (3.94)	41.34 (3.76)	<0.001
PLT (10^9^/L)	211.00 (167.00–253.00)	221.00 (185.00–265.00)	201.00 (156.75–246.00)	0.012	221.00 (185.00–281.00)	210.50 (165.00–250.00)	0.151
HALP	43.66 (33.09–61.47)	33.82 (24.15–40.83)	51.13 (38.06–66.49)	<0.001	25.59 (21.12–34.41)	45.64 (35.28–62.80)	<0.001
Treatment sequential				0.666			1.000
CCRT	133 (44.19)	42 (43.30)	91 (44.61)		18 (43.90)	115 (44.23)	
SCRT	144 (47.84)	49 (50.52)	95 (46.57)		20 (48.78)	124 (47.69)	
RT only	24 (7.97)	6 (6.19)	18 (8.82)		3 (7.32)	21 (8.08)	
RT modality				0.905			0.758
Definity RT	151 (50.17)	49 (50.52)	102 (50.00)		19 (46.34)	132 (50.77)	
Postoperative RT	109 (36.21)	36 (37.11)	73 (35.78)		15 (36.59)	94 (36.15)	
Palliative RT	41 (13.62)	12 (12.37)	29 (14.22)		7 (17.07)	34 (13.08)	
BED (Gy)	72.00 (72.00–72.00)	72.00 (72.00–72.00)	72.00 (72.00–72.00)	0.948	72.00 (72.00–72.00)	72.00 (72.00–72.00)	0.758
PTV (cc)	323.86 (257.00–394.24)	332.00 (289.00–453.00)	319.50 (233.62–378.85)	0.002	351.15 (289.00–453.00)	320.00 (246.25–389.00)	0.042
V5 (%)	44.00 (39.00–50.00)	50.00 (45.00–55.00)	41.00 (36.00–48.00)	<0.001	54.00 (48.00–58.00)	42.00 (38.00–49.25)	<0.001
V20 (%)	19.00 (16.00–22.00)	22.00 (19.00–25.00)	19.00 (16.00–20.00)	<0.001	23.00 (20.00–25.00)	19.00 (16.00–21.00)	<0.001
MLD (Gy)	11.00 (10.00–12.00)	12.00 (11.00–14.00)	10.00 (9.00–12.00)	<0.001	13.00 (12.00–14.00)	11.00 (9.75–12.00)	<0.001
Chemotherapy regimen				0.853			0.837
Paclitaxel + carboplatin/cisplatin	121 (40.20)	41 (42.27)	80 (39.22)		19 (46.34%)	102 (39.23%)	
Pemetrexed + carboplatin/cisplatin	105 (34.88)	33 (34.02)	72 (35.29)		12 (29.27%)	93 (35.77%)	
Etoposide + carboplatin/cisplatin	51 (16.94)	17 (17.53)	34 (16.67)		7 (17.07%)	44 (16.92%)	
None	24 (7.97)	6 (6.19)	18 (8.82)		3 (7.32%)	21 (8.08%)	
ICI				0.636			0.870
No	227 (75.42)	71 (73.20)	156 (76.47)		30 (73.17)	197 (75.77)	
Yes	74 (24.58)	26 (26.80)	48 (23.53)		11 (26.83)	63 (24.23)	

Data were expressed as n (%) or median (25th–75th percentile).

AC, adenocarcinoma; ALB, albumin; ALC, absolute lymphocyte count; BED, biologically effective dose; BMI, body mass index; CCRT, concurrent chemoradiotherapy; ECOG PS, Eastern Cooperative Oncology Group performance status; HALP, hemoglobin, albumin, lymphocyte and platelet; Hb, hemoglobin; ICI, immune checkpoint inhibitors; ILD, interstitial lung disease; MLD, mean lung dose; PLT, platelet; PTV, planning target volume; RT, radiotherapy; SCC, squamous cell carcinoma; SCLC, small cell lung cancer; SCRT, sequential chemoradiotherapy; Vx, the percentage of the lung volume that received more than x Gy.

Additionally, 95 patients were selected for the temporal validation cohort. Except for MLD and V5 , no significant differences were observed in the variables between the temporal validation cohort and the development cohort (**Table 2**).

**Table 2:** Baseline characteristics of patients in the development and validation cohorts.

Variables	Overall (n = 396)	Development cohort (n = 301)	Validation cohort (n = 95)	*P*
Gender				1.000
Female	101 (25.51)	77 (25.58)	24 (25.26)	
Male	295 (74.49)	224 (74.42)	71 (74.74)	
Age (years)	67.50 (61.00–72.00)	67.00 (60.00–72.00)	68.00 (63.00–72.00)	0.216
BMI (kg/m²)	23.44 (21.25–25.52)	23.44 (21.22–25.47)	23.78 (21.51–25.91)	0.330
ECOG PS				0.971
0	133 (33.59)	102 (33.89)	31 (32.63)	
1	182 (45.96)	138 (45.85)	44 (46.32)	
2	81 (20.45)	61 (20.27)	20 (21.05)	
Smoking				0.069
No	180 (45.45)	145 (48.17)	35 (36.84)	
Yes	216 (54.55)	156 (51.83)	60 (63.16)	
ILD				0.128
No	357 (90.15)	267 (88.70)	90 (94.74)	
Yes	39 (9.85)	34 (11.30)	5 (5.26)	
Emphysema				0.813
No	281 (70.96)	215 (71.43)	66 (69.47)	
Yes	115 (29.04)	86 (28.57)	29 (30.53)	
Histology				0.768
SCC	141 (35.61)	108 (35.88)	33 (34.74)	
AC	153 (38.64)	119 (39.53)	34 (35.79)	
SCLC	88 (22.22)	64 (21.26)	24 (25.26)	
Other	14 (3.54)	10 (3.32)	4 (4.21)	
Clinical stage				0.955
II	130 (32.83)	99 (32.89)	31 (32.63)	
III	162 (40.91)	122 (40.53)	40 (42.11)	
IV	104 (26.26)	80 (26.58)	24 (25.26)	
ALC (10^9^/L)	1.80 (1.50–2.19)	1.78 (1.49–2.23)	1.80 (1.60–2.07)	0.345
Hb (g/L)	122.29 (111.75–133.00)	122.00 (111.00–134.00)	122.00 (112.00–133.00)	0.877
ALB (g/L)	40.80 (38.00–43.73)	40.90 (38.00–43.60)	40.50 (37.70–44.10)	0.830
PLT (10^9^/L)	211.00 (167.00–252.00)	211.00 (167.00–253.00)	211.00 (169.00–251.00)	0.938
HALP	43.97 (32.74–61.69)	43.66 (33.09–61.47)	43.98 (32.06–63.13)	0.652
Treatment sequential				0.615
CCRT	180 (45.45)	133 (44.19)	47 (49.47)	
SCRT	184 (46.46)	144 (47.84)	40 (42.11)	
RT only	32 (8.08)	24 (7.97)	8 (8.42)	
RT modality				0.050
Definity RT	203 (51.26)	151 (50.17)	52 (54.74)	
Postoperative RT	132 (33.33)	109 (36.21)	23 (24.21)	
Palliative RT	61 (15.40)	41 (13.62)	20 (21.05)	
Chemotherapyregimen				0.628
Paclitaxel + platinum	160 (40.40)	121 (40.20)	39 (41.05)	
Pemetrexed + platinum	133 (33.59)	105 (34.88)	28 (29.47)	
Etoposide + platinum	68 (17.17)	51 (16.94)	17 (17.89)	
None	35 (8.84)	24 (7.97)	11 (11.58)	
BED (Gy)	72.00 (72.00–72.00)	72.00 (72.00–72.00)	72.00 (72.00–72.00)	0.226
PTV (cc)	322.62 (257.00–394.68)	323.86 (257.00–394.24)	322.00 (258.00–373.50)	0.078
V5 (%)	44.00 (38.00–50.00)	44.00 (39.00–50.00)	44.00 (37.50–50.00)	0.042
V20 (%)	19.00 (16.00–22.00)	19.00 (16.00–22.00)	19.00 (16.00–20.50)	0.092
MLD (Gy)	11.00 (10.00–12.00)	11.00 (10.00–12.00)	10.00 (10.00–11.65)	0.011
ICI				1.000
No	299 (75.51)	227 (75.42)	72 (75.79)	
Yes	97 (24.49)	74 (24.58)	23 (24.21)	

Data were expressed as n (%) or median (25th–75th percentile).

AC, adenocarcinoma; ALB, albumin; ALC, absolute lymphocyte count; BED, biologically effective dose; BMI,body mass index; CCRT, concurrent chemoradiotherapy; ECOG PS, Eastern Cooperative Oncology Group performance status; HALP, hemoglobin, albumin, lymphocyte and platelet; Hb, hemoglobin; ICI, immune checkpoint inhibitors; ILD, interstitial lung disease; MLD, mean lung dose; PLT, platelet; PTV, planning target volume; RP, radiation pneumonitis; RT, radiotherapy; SCC, squamous cell carcinoma; SCLC, small cell lung cancer; SCRT, sequential chemoradiotherapy; Vx, the percentage of the lung volume that received more than x Gy.

### 3.2 LASSO regression and multivariate logistic analysis

**Figure 2:**
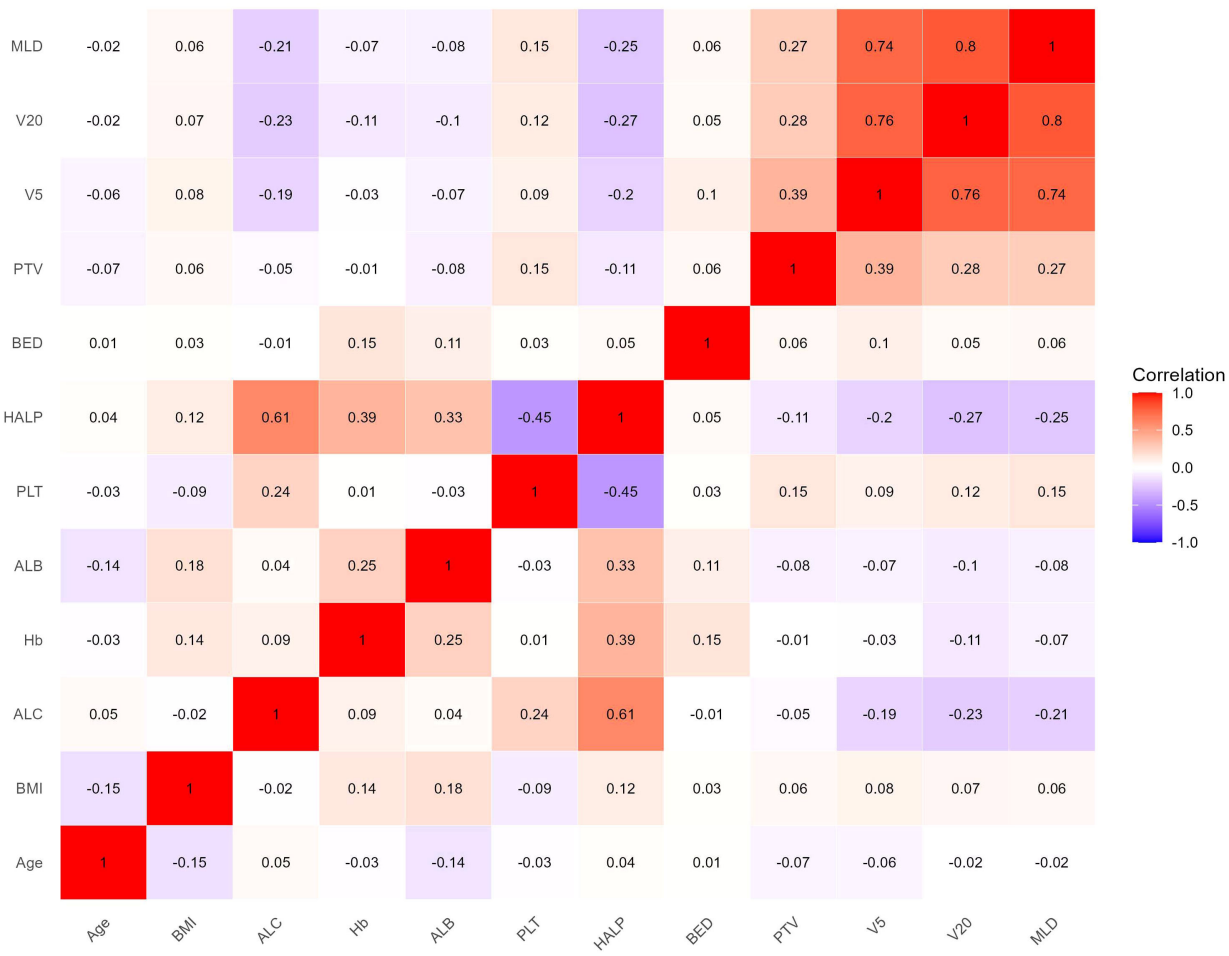
Correlation heat map of continuous variables. Color depth represents correlation coefficient (*r* value).

**Figure 3:**
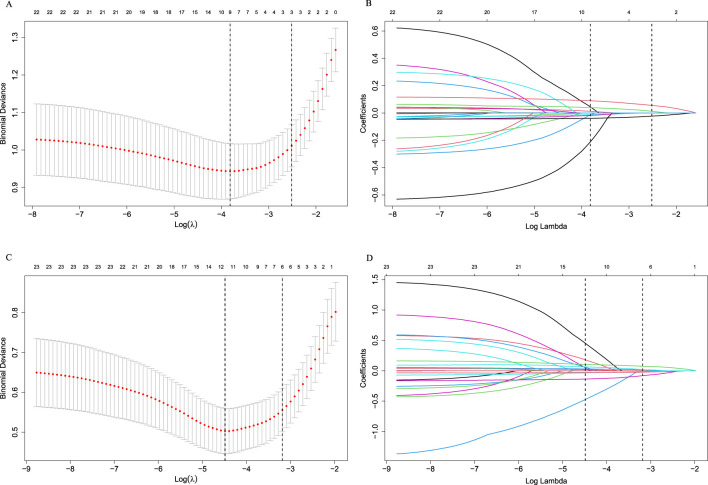
Selection of variables using the LASSO regression in the development cohort. **(A)** Selection of factors for symptomatic RP; **(B)** LASSO coefficients for symptomatic RP; **(C)** Selection of factors for severe RP; **(D)** LASSO coefficients for severe RP. LASSO cross-validation curves (10-fold cross-validation) for identifying optimal λ values. The best-matching factors were selected using the lambda.1se criterion (right dotted line). Selected λ values: 0.08095848 for symptomatic RP and 0.04146035 for severe RP.

The heat map indicated significant correlations among some continuous variables (**Figure 2**). To address multicollinearity and optimize model performance, LASSO regression was employed for variable selection. Using the lambda.1se criterion, LASSO analysis identified three variables (HALP, V5, and V20) out of the 27 variables as the most predictive features for symptomatic RP. For severe RP, six variables (Hb, ALB, HALP, V5, V20, and MLD) were selected (**Figure 3**).

Multivariate logistic analysis revealed that V5 and HALP were independent predictive factors for symptomatic RP (**Table 2**). For severe RP, ALB, Hb, HALP, and V5 emerged as independent predictors (**Table 3**). No significant multicollinearity was observed within the final models (all VIF <3) (**Table 3**). The Box-Tidwell test confirmed that all continuous predictors exhibited a linear relationship with the log-odds of the outcome , validating the linearity assumption of the logistic regression models and ensuring the statistical approach was appropriate.

**Table 3:** Multivariate logistic regression analysis for symptomatic and severe RP in the development cohort.

Variables	Symptomatic RP			Severe RP		
	OR (95% CI)	*P*	VIF	OR (95% CI)	*P*	VIF
Hb (g/L)	–	–	–	0.96 (0.92–0.99)	0.017	1.10
ALB (g/L)	–	–	–	0.84 (0.74–0.94)	0.004	1.08
HALP	0.95 (0.93–0.96)	<0.001	1.04	0.95 (0.91–0.98)	0.007	1.15
V5 (%)	1.11 (1.06–1.18)	<0.000	1.58	1.14 (1.05–1.25)	0.002	1.88
V20 (%)	1.06 (0.96–1.18)	0.259	1.59	1.04 (0.87–1.25)	0.641	2.03
MLD (Gy)	–	–	–	1.10 (0.78–1.53)	0.596	1.84

ALB, albumin; HALP, hemoglobin, albumin, lymphocyte, and platelet; Hb, hemoglobin; MLD, mean lung dose; RP, radiation pneumonitis; VIF, variance inflation factor; Vx, the percentage of the lung volume that received more than x Gy.

The HALP score achieve optimal diagnostic performance With an AUROC value of 0.77 (95% CI: 0.72–0.83) for symptomatic RP and 0.83 (95% CI: 0.76–0.90) for severe RP, at threshold of 40.85 (sensitivity: 71%, specificity: 75%) for symptomatic RP, and 34.53 (sensitivity: 76% , specificity: 77%) for severe RP.

### 3.3 Establishment and validation of the nomogram for symptomatic RP

**Figure 4:**
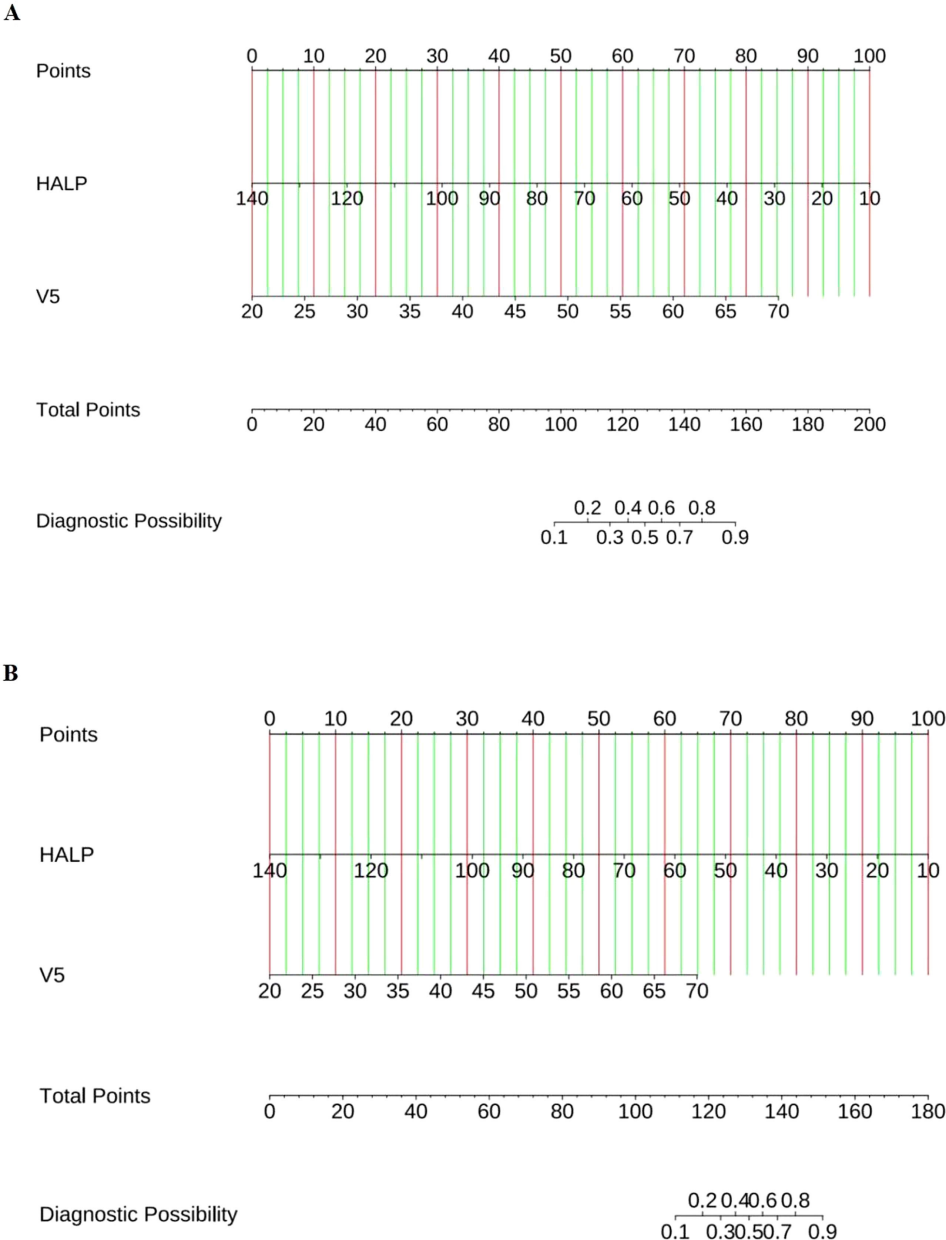
Nomogram for predicting the risk of RP. **(A)** Symptomatic RP; **(B)** Severe RP. Locate each variable’s score on the top “Points” axis; Sum all scores to obtain the “Total Points”; Read the predicted probability on the bottom axis.

**Figure 5:**
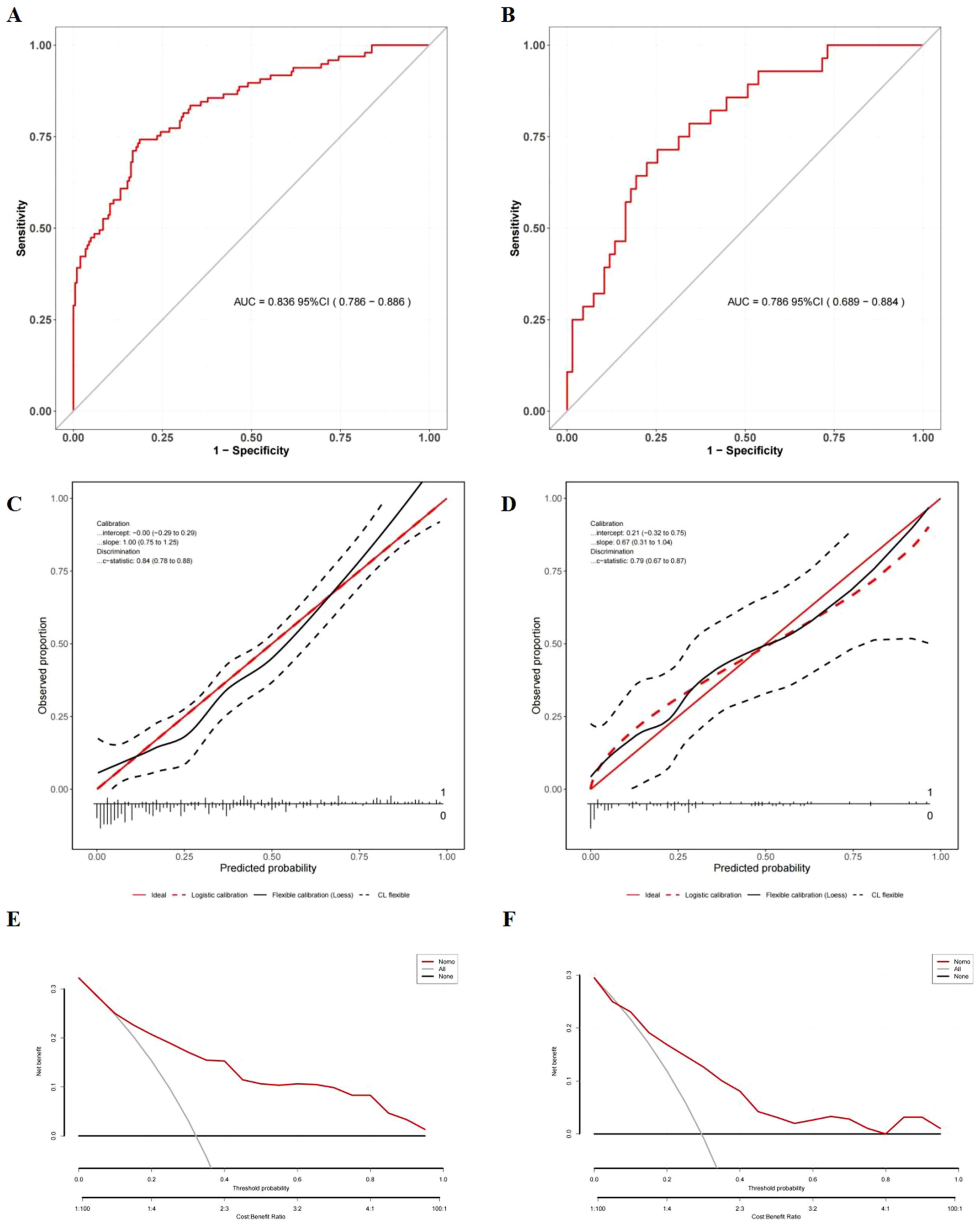
Evaluation of the nomogram model for symptomatic RP prediction. **(A)** ROC curves in the development cohort; **(B)** ROC curves in the validation cohort; **(C)** Calibration plots in the development cohort; **(D)** Calibration plots in the validation cohort; The x-axis is the nomogram-predicted probability, and the y-axis is the actual conversion rate. The red solid line indicates perfect agreement between predicted and actual probabilities. The red dotted line shows the nomogram’s performance, closer alignment with the red solid line signifies better predictive accuracy. **(E)** DCA in the development cohort; **(F)** DCA in the validation cohort; DCA illustrates the expected net benefit per patient based on the nomogram’s prediction of symptomatic radiation pneumonitis (RP) risk. The solid horizontal line corresponds to the scenario where no patients have symptomatic RP (the “none” strategy), while the red line represents the prediction by the nomogram (“Nomo”). The gray line likely corresponds to the “all” strategy (all patients considered). As the curve of the nomogram extends, the net benefit changes.

Based on the results of multivariate logistic regression, a nomogram incorporating HALP and V5 was constructed (**Figure 4A**, **Supplementary Table S2**). The HALP-V5 composite model exhibited higher discriminative ability than any single variable, with an area under the receiver operating characteristic curve (AUROC) of 0.84 (95% confidence interval [CI]: 0.79–0.89) (**Figure 5A**).

Internal validation using the bootstrap resampling method (1,000 iterations) demonstrated the model’s stability, yielding a comparable AUROC of 0.89 (95% CI: 0.86–0.93). Although the AUROC (0.79; 95% CI: 0.69–0.88) of the temporal validation cohort (**Figure 5B**) was lower than that of the development cohort, DeLong’s test did not identify a significant difference in the AUROC between the two models (*P* = 0.376). This finding suggests that the temporal validation model achieved consistent performance, further confirming the model’s good reproducibility

Calibration was rigorously assessed using the Hosmer-Lemeshow test and calibration plots. In both the development (χ² = 12.70, *P* = 0.123) and validation (χ² = 9.862, *P* = 0.274) cohorts, no significant discrepancies were observed between the predicted and observed probabilities of RP. Visual inspection of the calibration plots further demonstrated strong alignment, with data points clustering closely around the ideal 45-degree line, validating the model’s reliability (**Figures 5C, D**).

DCA showed that the nomogram offered significantly greater net clinical benefit than “universal intervention” or “no-intervention” strategies across a wide range of threshold probabilities: 9.0%–99.0% in the development cohort (**Figure 5E**) and 7.0%–98.0% in the validation cohort (**Figure 5F**). These findings underscore the monogram’s robust performance and clinical utility for risk stratification.

### 3.4 Establishment and validation of the nomogram for severe RP

**Figure 6:**
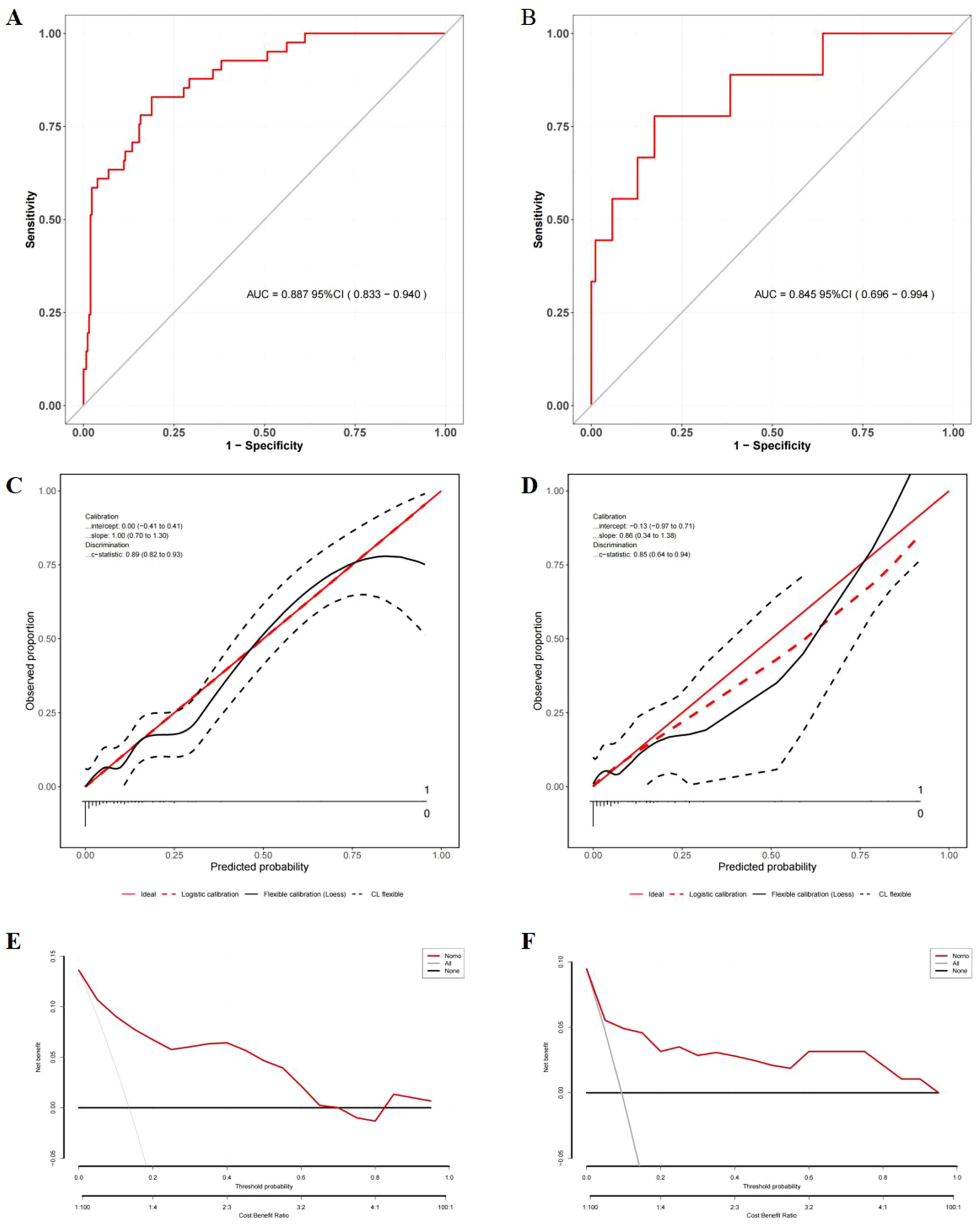
Evaluation of the nomogram model for the prediction of severe RP. **(A)** ROC curves in the development cohort; **(B)** ROC curves in the validation cohort; **(C)** Calibration plots in the development cohort; **(D)** Calibration plots in the validation cohort; **(E)** DCA in the development cohort; **(F)** DCA in the validation cohort.

The nomogram integrating HALP-V5 to predict grade ≥3 RP achieved an AUROC of 0.89 (95% CI: 0.83–0.94) in the development cohort (**Figures 4B**, **6A**, **Supplementary Table S2**), which outperformed the model for symptomatic RP (AUROC 0.84; 95% CI: 0.79–0.89).

Comprehensive validation confirmed the model’s robust performance. In the temporal validation cohort, the nomogram exhibited good discrimination with an AUROC of 0.85 (95% CI: 0.70–0.99) (**Figure 6B**), showing no significant difference compared to the development cohort (DeLong’s test, *P* = 0.606).

Strong calibration was observed in the model, as evidenced by the Hosmer-Lemeshow test (development cohort: χ² = 11.026, *P* = 0.200; validation cohort: χ² = 9.048, *P* = 0.338) and calibration plots (**Figures 6C, D**).

DCA demonstrated the nonogram’s good clinical utility compared to blanket treatment strategies across threshold probability of 2.0%–68.0% for the development cohort and 2.0%–96.0% for the validation cohort (**Figures 6E, F**).

### 3.5 Comparison of AUROC between HALP and dosimetric parameters

As shown in **Table 4**, comparisons of AUROC of the nomogram with those of individual prognostic factors revealed that the comprehensive model exhibited higher discrimination than any single variable both in symptomatic and severe RP. The predictive ability of the HALP score is superior to its individual components and comparable to dosimetry parameters (V5, V20, MLD, and PTV), indicating that its predictive ability was not inferior to that of dosimetric parameters.

**Table 4:** Comparison of predictive variables between nutritional and dosimetric parameters for symptomatic and severe RP in the development cohort.

Variables	Symptomatic RP				Severe RP			
	AUC (95% CI)	Sensitivity	Specificity	Cutoff	AUC (95% CI)	Sensitivity	Specificity	Cutoff
ALC (10^9^/L)	0.69 (0.63–0.76)	0.28	0.37	1.77	0.74 (0.66–0.82)	0.20	0.40	1.73
Hb (g/L)	0.65 (0.58–0.71)	0.40	0.31	118.50	0.73 (0.65–0.81)	0.20	0.37	119.50
ALB (g/L)	0.63 (0.56–0.70)	0.48	0.29	39.45	0.75 (0.67–0.84)	0.32	0.24	38.45
PLT (10^9^/L)	0.59 (0.52–0.66)	0.82	0.37	175.50	0.57 (0.47–0.67)	0.78	0.36	181.50
HALP	0.77 (0.72–0.83)	0.71	0.75	40.85	0.83 (0.76–0.90)	0.76	0.77	34.53
PTV (cc)	0.61 (0.54–0.68)	0.95	0.24	228.24	0.60 (0.51–0.69)	0.98	0.20	228.24
V5 (%)	0.76 (0.70–0.82)	0.60	0.82	49.50	0.81 (0.74–0.89)	0.73	0.75	49.50
V20 (%)	0.75 (0.69–0.82)	0.57	0.83	21.50	0.81 (0.75–0.87)	0.88	0.57	19.50
MLD (Gy)	0.73 (0.67–0.80)	0.69	0.69	11.50	0.80 (0.73–0.86)	0.80	0.63	11.50
Nomogram	0.84 (0.79–0.89)	0.74	0.84	0.39	0.89 (0.83–0.94)	0.83	0.81	0.14

ALB, albumin; ALC, absolute lymphocyte count; HALP, hemoglobin, albumin, lymphocyte, and platelet; Hb, hemoglobin; MLD, mean lung dose; PLT, platelet; PTV, planning target volume; RP, radiation pneumonitis; Vx, the percentage of the lung volume that received more than x Gy.

## 4 Discussion

Despite considerable efforts over the past decades, no validated biomarkers or robust prediction models have been clinically adopted for RP (4, 12), which underscores an urgent need for reliable predictive biomarkers in radiation oncology. Recent studies have explored the predictive value of inflammatory and nutritional biomarkers derived from routine blood tests, such as neutrophils, ALB, ALC, Hb, PLT, neutrophil-to-lymphocyte ratio, monocyte-to-lymphocyte ratio, and platelet-to-lymphocyte ratio in RP risk assessment (16, 19–21, 23, 25, 26). These findings highlight the potential of routine blood-based inflammatory-nutritional biomarkers for predicting RP development. Since its introduction, the HALP score has emerged as a prognostic indicator for various cancers (27, 28). However, its predictive value for RP remains understudied. Our study demonstrates that HALP can serve as a promising tool for identifying RP risk, suggesting that elevated inflammation and poor nutritional status are associated with increased RP risk. This discovery underscores the clinical potential of pre-radiotherapy interventions targeting nutritional optimization and inflammatory modulation to forestall RP development.

The predictive capacity of HALP for RP can be interpreted through its components, where HALP is calculated from Hb, ALB, ALC, and PLT in peripheral blood (18). Serum ALB and Hb, as fundamental plasma proteins with diverse physiological functions, have been established as reliable prognostic indicators across various cancers (29, 30). While ALB has demonstrated prognostic value for radiotherapy-related toxicities (17), its predictive utility for RP remains controversial (16, 31). Similarly, conflicting evidence exists regarding the correlation between Hb and RP risk (16, 23). Our study revealed that Hb and ALB exhibited acceptable AUROC for both symptomatic and severe RP (AUROC >0.6). Thus, their predictive role should be acknowledged, and further investigation is warranted. ALC, another component of the HALP score, exhibits a close pathophysiological relationship with RP. Lymphopenia, affecting approximately 70% of thoracic cancer patients receiving radiotherapy (32), is linked with a poor prognosis (19, 33). Although radiation-induced lymphocytic alveolitis has been consistently documented (34), current evidence regarding ALC’s role in RP prediction presents conflicting perspectives. While some studies, including animal experiments, suggest an inverse correlation between lymphocyte counts and RP risk (19, 20), others show no significant intergroup differences in lymphocyte levels between RP and non-RP groups (25, 26). Our results tentatively support reduced ALC levels as a risk factor for RP, though this association did not achieve statistical independence. Notably, the limited AUROC values (ALB: 0.63; ALC: 0.69; Hb: 0.61) caution against overinterpreting the clinical application value of these individual biomarkers. Contrary to previous reports linking thrombocytopenia to elevated RP risk (22, 35), our analysis failed to demonstrate a significant association between PLT and RP.

Considering the current evidence regarding the predictive roles of its components , the predictive capability of the HALP score for RP is easily understandable. The superior predictive performance of the HALP score compared to its individual components (AUROC: 0.77 versus 0.59–0.69) highlights the clinical advantage of multidimensional inflammation-nutrition assessments over unidimensional parameter analysis.

The relationship among inflammation, RP, and nutritional status is intricate and interactive. Ionizing radiation triggers inflammatory reactions, which contribute to tissue remodeling and subsequent fibrosis (6). In this respect, RP can be regarded as acute lung tissue inflammation resulting from radiation exposure (4, 6, 36). Inflammatory conditions can exacerbate disease-related malnutrition and impair nutritional intervention response. Conversely, nutrition also influences the body’s inflammatory response (37). Malnutrition is known to worsen treatment-related toxicities (e.g., mucositis, esophagitis) and diminish radiotherapeutic efficacy (15–17). Most radiotherapy-related toxicities frequently exacerbate nutritional deficits and contribute to malnutrition (38). Given the current understanding of the interactions among radiotherapy, inflammation, and RP, the utility of inflammatory-nutritional biomarkers to predict RP risk may vary significantly. Numerous inflammatory cytokines have been reported to be associated with RILI development (8, 39). However, most of these studies are limited to experimental research and lack clinical significance. Indeed, no clinically implementable biomarker, including cytokines, proteins, or other serum markers, has been standardized for RP prediction in routine practice (6).

The impact of nutritional status on RP risk remains poorly studied. Only one study, which used the subjective general assessment (SGA) as a nutritional indicator, indicated that SGA was a significant predictor of RP in lung cancer patients (13). However, SGA is subjective and lacks quantitative biochemical measurements, making it prone to bias and yielding unreliable results. In contrast, our analysis leverages objective hematological parameters, allowing for a more accurate and comprehensive assessment of the predictive value of nutritional status in RP development.

As an inflammatory-nutritional indicator, the HALP score may be the first to focus on its predictive value for RP, although its importance has been widely reported in the prognosis of various cancers. Notably, the HALP score demonstrated better predictive discrimination compared with some major dosimetric parameters, including V5, V20, and MLD, which have well-established correlations with RILI development (4, 7, 24). A recent meta-analysis showed that machine learning models incorporating multimodal characteristics exhibit 75% accuracy in predicting moderate-to-severe RP (40). Notably, our HALP-V5 model demonstrated better predictive performance for RP (AUROC >0.8), with good reproducibility and predictive accuracy. Across a broad range of threshold probabilities for symptomatic RP (**Figure 5E**), the DCA demonstrates that the HALP-V5 model serves as a dependable tool for its full-cycle clinical management. However, the clinical utility of severe RP is suboptimal from DCA results in development cohort (**Figure 5F**), possibly due to the low number of RP patients and outliers.

Compared with the previously developed models incorporating multi-modal features (10, 11, 40), the components of HALP-V5 model can be easily calculated from pre-radiotherapy routine assessment parameters (HALP and V5) without any additional examinations. From a clinical translation perspective, the predictive value of the HALP score and the HALP-V5 composite model for RP has certain clinical application potential. According to the cutoff of the model, patients in high-risk group (HALP <40.85 or V5 >49.5%) were more likely to develop symptomatic RP compared with those in low-risk group. By accurately estimating the probability of RP through the HALP-V5 model, clinicians should pay more attention to variations in clinical symptoms and chest CT scans and rationally adjust the radiation dose and fractionation regimens before radiotherapy implementation, thereby reducing the risk of concurrent RP. The nomogram facilitates visual evaluation but is not suitable for direct calculation of RP predicted probability; thus, we provide a calculation formula to address this limitation (**Supplementary Table S2**). Here we use an example to illustrate the formula: assuming that a patient has a HALP of 50 and a V5 of 50%, the corresponding probability for symptomatic RP is 37.44%.

Although current evidence for routine nutritional intervention in lung cancer is limited (41), our findings support the recommendation for nutritional education and counseling prior to radiotherapy to prevent RP. Improving nutritional status may be a relatively simple and low-cost way to control RP in radiotherapy patients compared with other contributing RP factors. Further research is needed into the potential of nutritional interventions in reducing the incidence of RILI in malnourished patients receiving radiotherapy.

However, several limitations should be noted when interpreting our findings. First, the inherent limitations of the single-center retrospective nature with a small sample size make the study susceptible to selection and information bias, depressing statistical power and rendering assessment difficult. Second, the lack of spatial/domain external validation may hinder a comprehensive assessment of the model’s generalizability and overfitting risk, thereby undermining its practical application. Third, the multifactorial process of RP development (5–7) necessitates rigorous control of potential confounders. Unanalyzed confounders, particularly lung function, which has been shown to correlate with both nutritional status and the development of symptomatic RP (42, 43), may compromise the robustness of the results. Well-designed, multicenter prospective studies with sufficient sample sizes are needed to further validate our findings. The model should be continuously optimized and adjusted in consideration of clinical practice, such as patient individual differences and variations in radiation equipment, to ensure its clinical applicability and reliability in real-world settings.

## Conclusion

This study established the HALP score as an independent predictor for both symptomatic and severe RP, with superior predictive capability over dosimetric parameters. When HALP is integrated with V5, the HALP-V5 composite model achieves outstanding discriminative performance. Pre-radiotherapy calculation of the HALP score can be employed as a simple supplementary for predicting RP. Radiotherapy dose constraints should be imposed for high-risk patients (low HALP levels) while permitting dose escalation in low-risk cohort.

## Data Availability

The raw data supporting the conclusions of this article will be made available by the authors, without undue reservation.
