# Supplementary material for: Predictive significance of the hemoglobin, albumin, lymphocyte, and platelet score for radiation pneumonitis in lung cancer patients: a respective comparative study with dosimetric parameters
Source: Front Oncol. 2025 Jun 4;15:1605094. doi: 10.3389/fonc.2025.1605094 (PMC12177529; doi:10.3389/fonc.2025.1605094)
Supplement: Supplementary file 1 [file Table1.docx]

TABLE S1 Grading for radiation pneumonitis based on the Common Terminology Criteria for Adverse Events (CTCAE) 5.0.

| RTOG | CTCAE v. 5.0 |
| --- | --- |
| Grade 0 | No changes |
| Grade 1 | Asymptomatic or mild symptoms |
| Grade 2 | Moderate symptoms of pneumonitis (severe cough) and radiographic changes (radiographic patches) |
| Grade 3 | Severe symptoms of pneumonitis, dense radiographic changes |
| Grade 4 | Symptoms of severe respiratory failure requiring assisted ventilation or continuous O_2_ |
| Grade 5 | Death-related late effects of radiotherapy |
